# Supplementary material for: Directed evolution of compact RNA-guided nucleases for enhanced activity in mammalian cells
Source: Genome Biol. 2026 Jun 16;27:213. doi: 10.1186/s13059-026-04144-5 (PMC13330202; doi:10.1186/s13059-026-04144-5)
Supplement: Supplementary file 1 — Additional file 1. Contains supplementary figures. [file 13059_2026_4144_MOESM1_ESM.pdf]

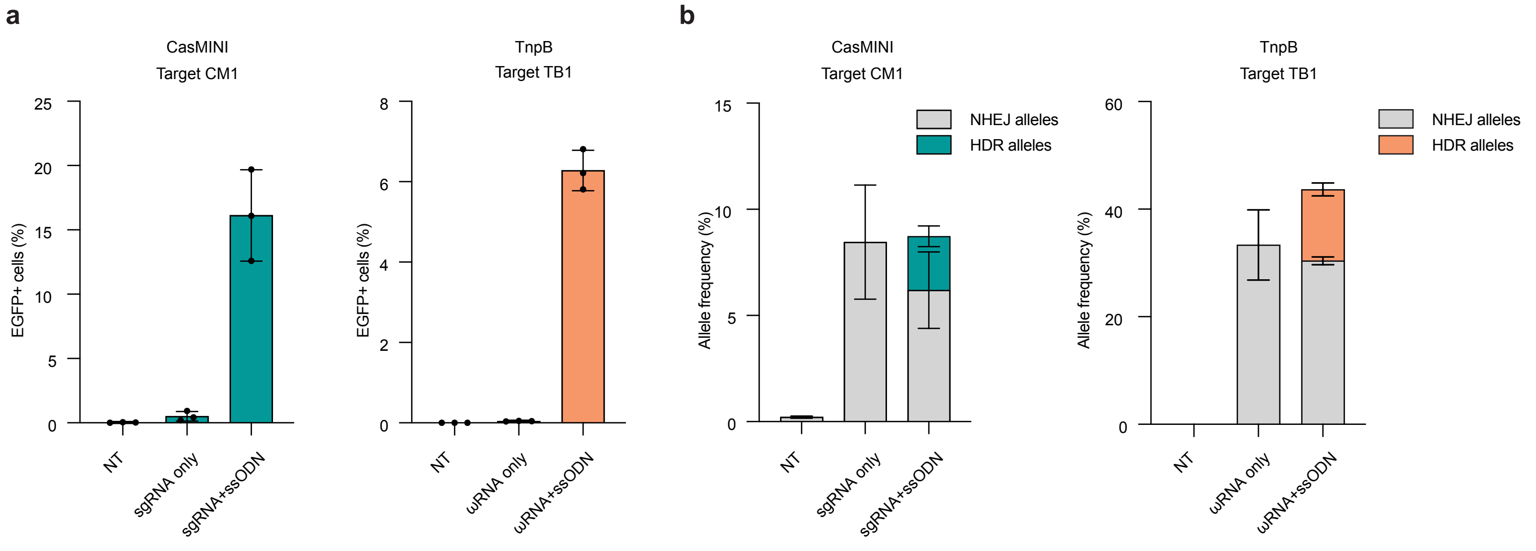

Fig. S1: *HDR reporter validation.* **a, b** Testing of CasMINI and TnpB genome editors at two targets (Target CM1 for CasMINI and Target TB1 for TnpB) within the HDR reporter framework in HEK293T cells (n = 3 biological replicates). EGFP percentages assessed by flow cytometry (**a**) and HDR and NHEJ allele percentages obtained through ampliconNGS (**b**) were used to quantify editing outcomes for indicated nucleases. NT, non-targeting. Each dot represents an individual biological replicate, and bars represent the mean ± standard deviation.

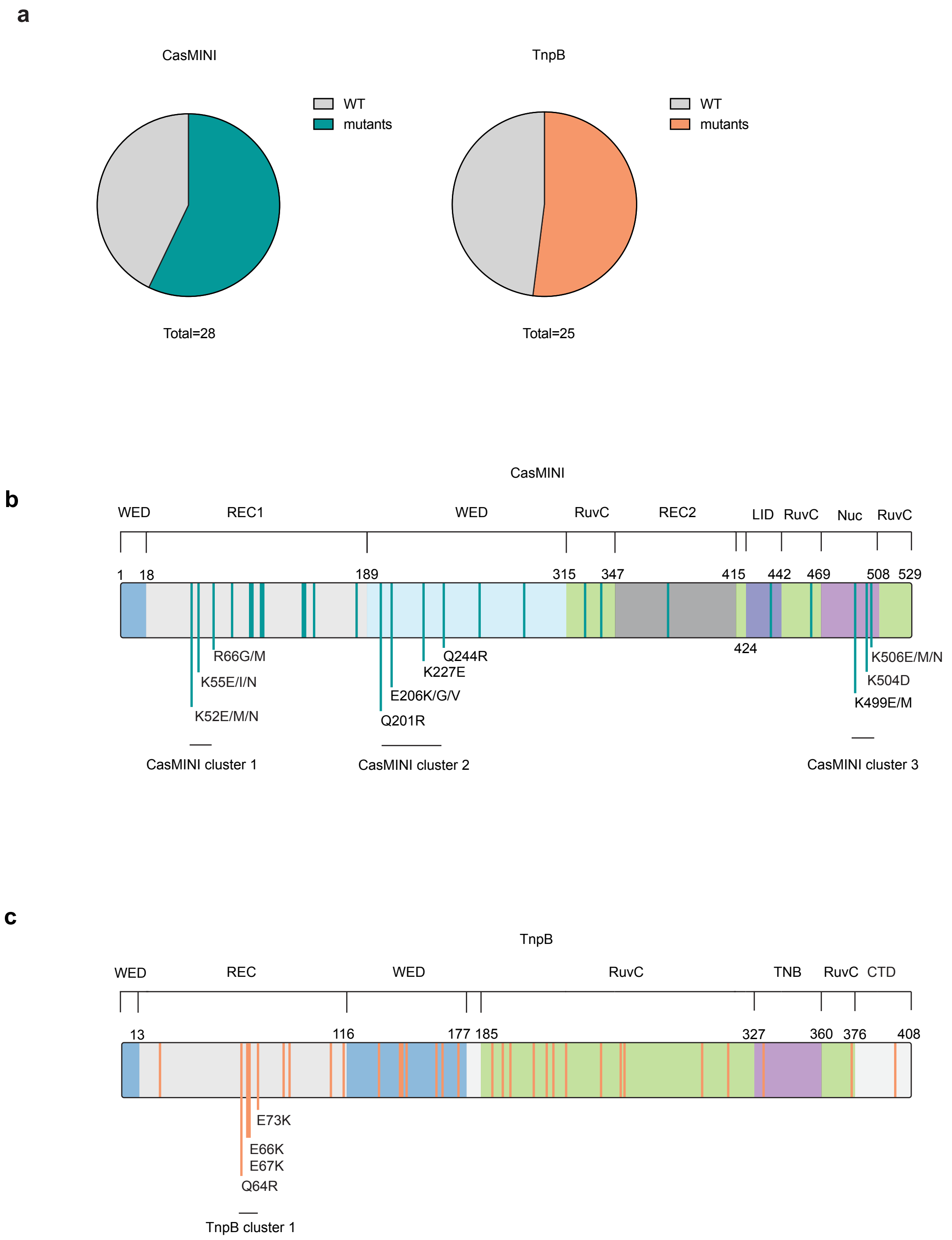

Fig. S2: *Localization of mutations enriched after selection for HDR with CasMINI and TnpB.* **a** Pie chart showing proportion of unmutated (WT) and mutated (variants) sequences in libraries used for the first round of selection for indicated nucleases. For purpose of estimating this proportion 28 (for CasMINI) or 25 (for TnpB) randomly picked colonies from respective libraries were picked and analyzed by Sanger sequencing. **b, c** Mutations from Fig. 1d mapped on the domain organization of CasMINI (PDB: 7L49) (**b**) and TnpB (PDB: 8H1J) (**c**). The mutations from clusters of enriched mutations after round 4 of selection are labelled for each nuclease.

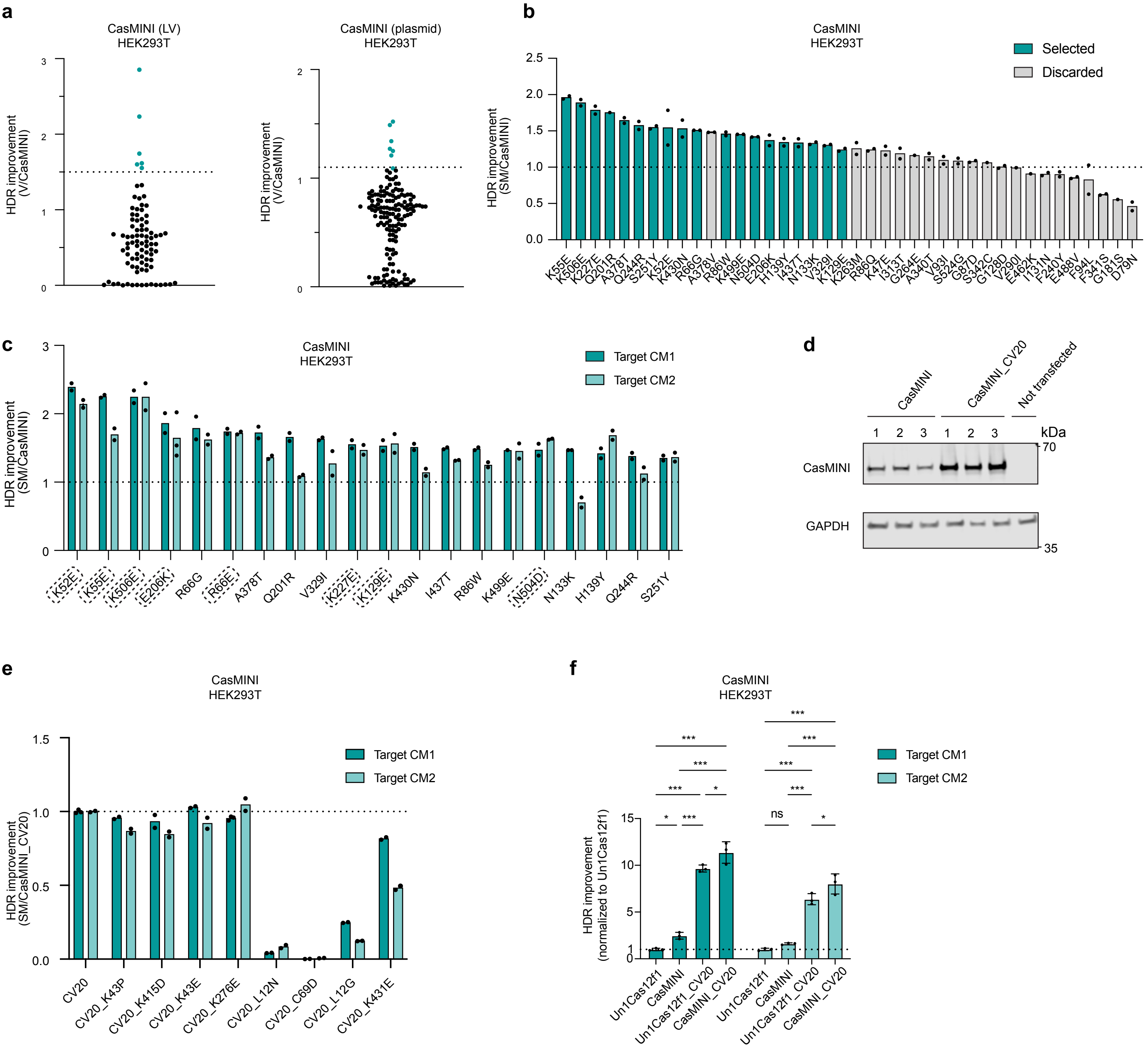

Fig. S3: *Selection of CasMINI mutations for generating combinatorial variants (CVs).* **a** HDR efficiencies of 88 CasMINI variants were tested in a lentiviral (LV) format and 158 CasMINI variants tested with plasmid delivery at Target CM1 in HEK293T cells. HDR values were measured as percentage of EGFP-positive cells from BFP/mCherry double positive cells (BFP marks the guide-ex-pressing construct; mCherry marks the nuclease-expressing construct) and normalized to CasMINI. Highlighted variants (teal) were selected for further validation using plasmid delivery in duplicate. **b** HDR efficiencies of single mutants (SMs) derived from selected high-performing CasMINI variants or enriched in NGS after round four of selection, tested at Target CM1 in HEK293T cells. HDR values were measured as percentage of EGFP-positive cells from BFP/mCherry double positive cells (BFP marks the guide-expressing construct; mCherry marks the nuclease-expressing construct) and normalized to CasMINI (*n* = 2 biological replicates). Highlighted SMs (teal) were selected for further testing at the orthogonal HDR reporter target (Target CM2). A378V was not selected due to the presence of A378T with similar or slightly higher editing efficiency, which was chosen for further analysis. Each dot represents an individual biological replicate, and bars represent the mean. **c** HDR efficiencies of selected CasMINI SMs tested in parallel at two distinct targets (Target CM1 and Target CM2) in HEK293T cells, measured as percentage of EGFP-positive cells from BFP/mCherry double positive cells (BFP marks the

guide-expressing construct; mCherry marks the nuclease-ex-pressing construct), with values normalized to CasMINI (*n* = 2 biological replicates). The mutations selected for making combinatorial variants are highlighted. Each dot represents an individual biological replicate, and bars represent the mean. **d** Western blot analysis of CasMINI and CasMINI_CV20 protein expression levels. Expression of each nuclease was performed in triplicate together with sgRNA for Target CM1 in HEK293T cells. GAPDH was used as a loading control. **e** HDR efficiencies of eight newly generated CasMINI variants, each containing one mutation derived from EVOLVEpro, cloned onto the CasMINI_CV20 background, tested in HEK293T cells (*n* = 2 biological replicates). HDR values were measured as percentage of EGFP-positive cells from BFP/mCherry double positive cells (BFP marks the guide-expressing construct; mCherry marks the nuclease-expressing construct) and normalized to the previously best-performing variant, CasMINI_CV20. Each dot represents an individual biological replicate, and bars represent the mean. **f** Comparison of editing efficiencies of Un1Cas12f1, CasMINI, Un1Cas12f1_CV20, CasMINI_CV20 at Target CM1 and Target CM2 in HEK293T cells. HDR values were measured as percentage of EGFP-positive cells from BFP/mCherry double positive cells (BFP marks the guide-expressing construct; mCherry marks the nuclease-expressing construct) and normalized to Un1Cas12f1 (*n* = 3 biological replicates). Each dot represents an individual biological replicate, and bars represent the mean ± standard deviation. All *P* values were calculated using an unpaired, two-sided t-test. ns, not significant ($P \geq 0.05$), *$P < 0.05$, **$P < 0.01$, ***$P < 0.001$.

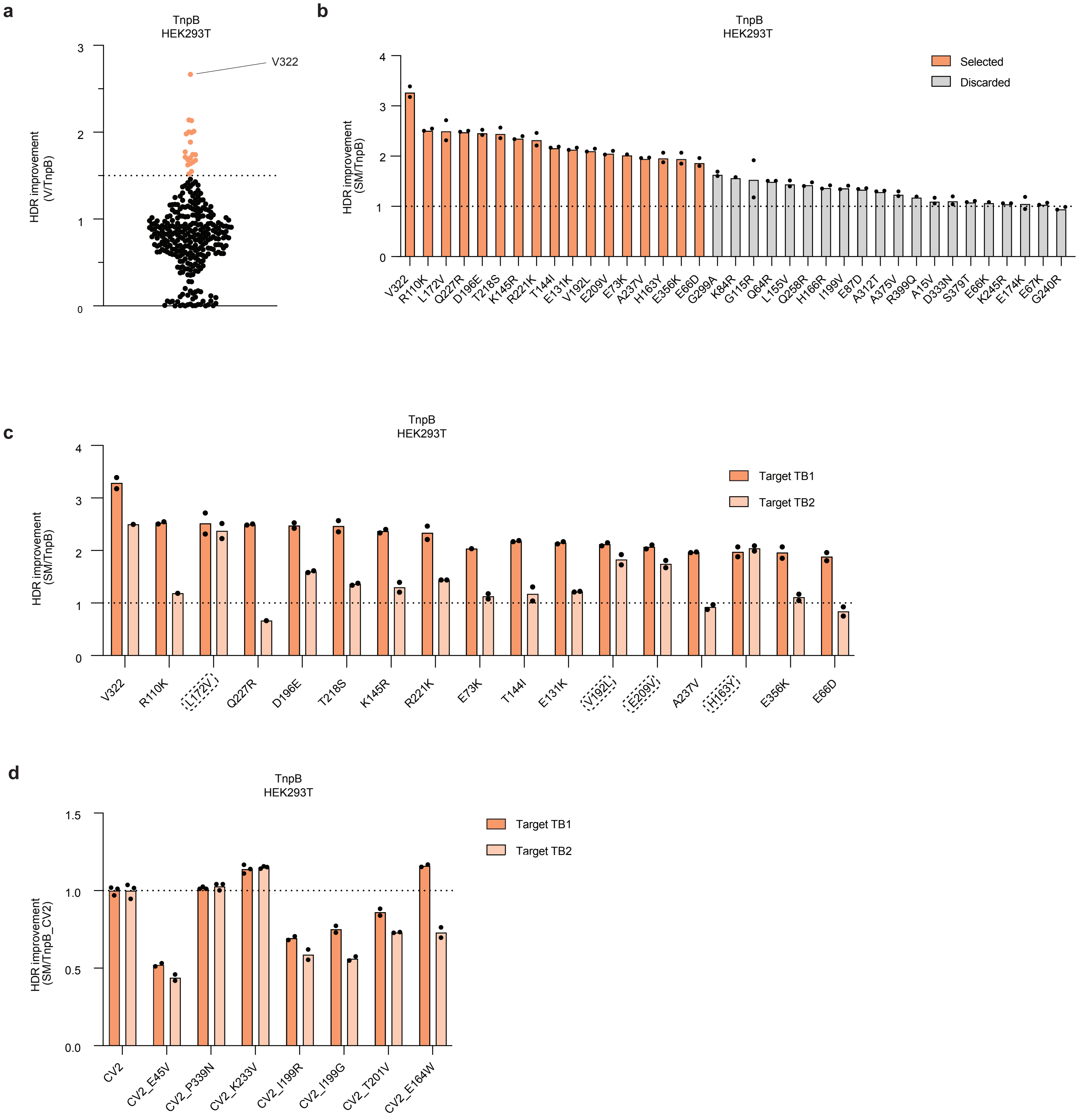

Fig. S4: *Selection of TnpB mutations for generating combinatorial variants (CVs).* ***a*** HDR efficiencies of 311 TnpB variants with plasmid delivery at Target TB1 in HEK293T cells. HDR values were measured as a percentage of EGFP-positive cells from BFP/mCherry double positive cells (BFP marks the guide-expressing construct; mCherry marks the nuclease-expressing construct) and normalized to TnpB. Highlighted variants (orange) were selected for further validation using plasmid delivery in duplicate. Top-performing is indicated (V322). **b** HDR efficiencies of all TnpB single mutants (SMs) derived from selected high-performing TnpB variants or enriched in NGS after round four of selection, tested at Target TB1 in HEK293T cells (*n* = 2 biological replicates). HDR values were measured as percentage of EGFP-positive cells from BFP/ mCherry double positive cells (BFP marks the guide-expressing construct; mCherry marks the nuclease-expressing

construct) and normalized to TnpB. Highlighted SMs were selected for further testing at the orthogonal HDR reporter target (Target TB2). Each dot represents an individual biological replicate, and bars represent the mean. **c** HDR efficiencies of selected TnpB SMs tested in parallel at two distinct targets (Target TB1 and Target TB2) in HEK293T cells measured as percentage of EGFP-positive cells from BFP/mCherry double positive cells (BFP marks the guide-expressing construct; mCherry marks the nuclease-expressing construct), with values normalized to TnpB (*n* = 2 biological replicates). The mutations selected for making combinatorial variants are highlighted. Each dot represents an individual biological replicate, and bars represent the mean. **d** HDR efficiencies of seven newly generated TnpB variants, each containing one mutation derived from EVOLVEpro, cloned onto the TnpB_CV2 background, tested in HEK293T cells (*n* = 2 biological replicates). HDR values were measured as percentage of EGFP-positive cells from BFP/mCherry double positive cells (BFP marks the guide-expressing construct; mCherry marks the nuclease-expressing construct) and normalized to the previously best-performing variant, TnpB_CV2. Each dot represents an individual biological replicate, and bars represent the mean.

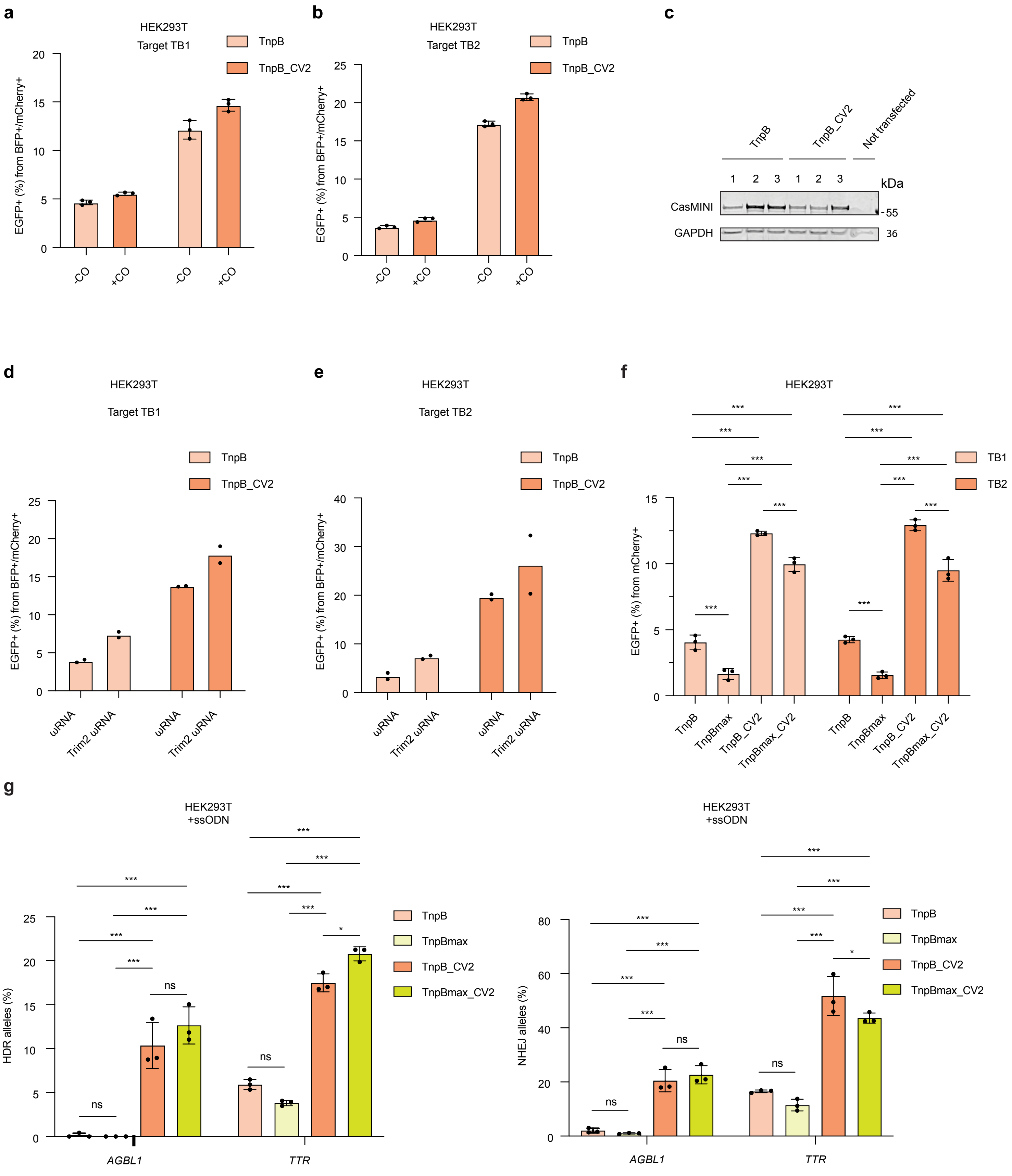

Fig. S5: *Mammalian codon-optimization and usage of shorter scaffold for TnpB enhance editing at HDR reporter targets in HEK293T cells.* **a, b** HDR efficiencies with mammalian codon-optimized (+CO) and not codon-optimized (-CO) versions of TnpB and TnpB_CV2 in HEK293T cells at HDR reporter Target TB1 (**a**) and Target TB2 (**b**) (*n* = 3 biological replicates). HDR was measured as percentage of EGFP-positive cells from BFP/mCherry double positive

cells (BFP marks the guide-expressing construct; mCherry marks the nuclease-expressing construct). Each dot represents an individual biological replicate, and bars represent the mean ± standard deviation. **c** Western blot analysis of mammalian codon-optimized TnpB and TnpB_CV2 protein expression levels (*n* = 3 biological replicates). Expression of each nuclease was performed together with ωRNA for Target TB1 in HEK293T cells. GAPDH was used as a loading control. **d, e** HDR efficiencies of mammalian codon-optimized TnpB and TnpB_CV2 combined with either Trim2 ωRNA scaffold or original ωRNA in HEK293T cells at HDR reporter Target TB1 (**d**) and Target TB2 (**e**). HDR was measured as percentage of EGFP-positive cells from BFP/mCherry double positive cells (BFP marks the guide-expressing construct; mCherry marks the nuclease-expressing construct) (*n* = 2 biological replicates). Each dot represents an individual biological replicate, and bars represent the mean. **f** HDR efficiencies of mammalian codon-optimized TnpB, TnpBmax, TnpB_CV2 and TnpBmax_CV2 combined with Trim2 ωRNA scaffold in HEK293T cells at HDR reporter targets TB1 and TB2. HDR was measured as percentage of EGFP-positive cells from mCherry+ cells (all-in-one construct was used for this experiment) (*n* = 3 biological replicates). **g** HDR and NHEJ efficiencies of mammalian codon-optimized TnpB, TnpBmax, TnpB_CV2 and TnpBmax_CV2 combined with Trim2 ωRNA scaffold in HEK293T cells at indicated endogenous sites. HDR and NHEJ were measured with ampliconNGS (*n* = 3 biological replicates). Each dot represents an individual biological replicate, and bars represent the mean ± standard deviation. All *P* values were calculated using an unpaired, two-sided t-test. ns, not significant ($P \geq 0.05$), *$P < 0.05$, **$P < 0.01$, ***$P < 0.001$.

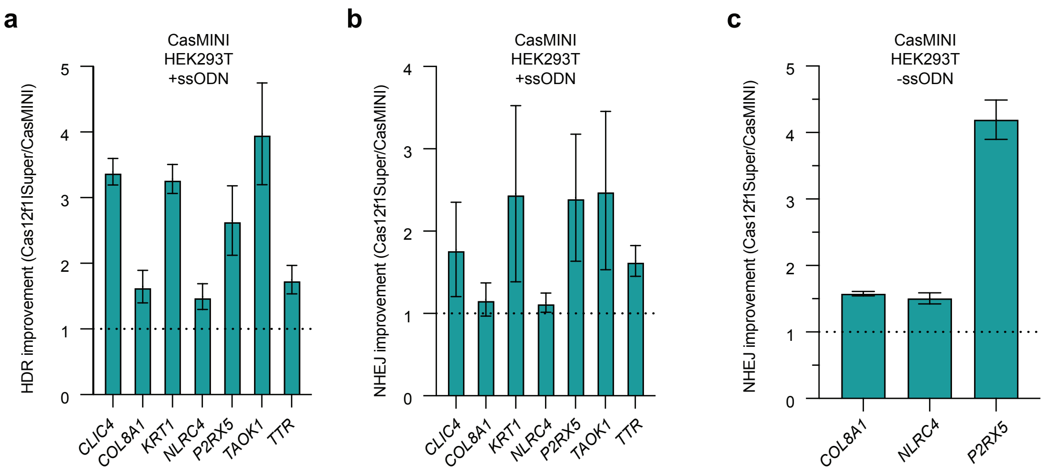

Fig. S6: *Improved variant Cas12f1Super edits endogenous genomic loci.* **a, b** Fold change of HDR efficiency (**a**) or NHEJ efficiency (**b**) of Cas12f1Super relative to CasMINI in presence of ssODN measured with ampliconNGS at indicated endogenous loci in HEK293T cells (*n* = 3 biological replicates). **c** Fold change of NHEJ efficiency of Cas12f1Super relative to CasMINI in absence of ssODN measured with ampliconNGS at indicated endogenous loci in HEK293T cells (*n* = 3 biological replicates). Bars represent mean ± standard deviation. Fold change was calculated from group means, with propagated standard deviation shown as error bars.

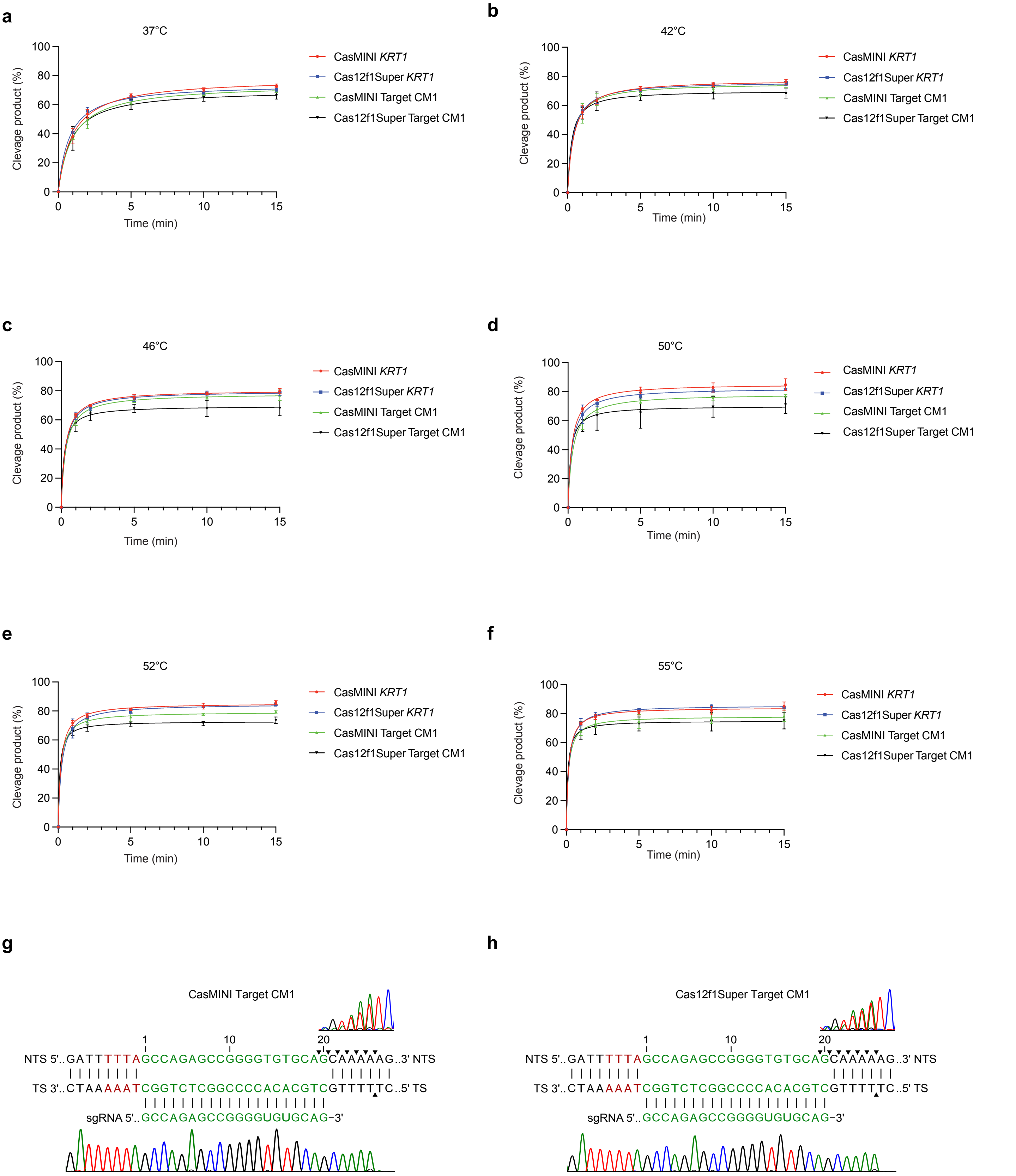

Fig. S7: *In vitro cleavage assays with CasMINI and Cas12f1Super.* **a-f** Percentages of fully cleaved substrates at two different targets at designated time points by CasMINI or Cas12f1Super (*n* = 3 biological replicates). Each dot represents the mean of three biological replicates ± standard deviation. **g, h** Run-off Sanger sequencing of the plasmid products cleaved by CasMINI (**g**) or Cas12f1Super (**h**). Cleavage positions at both the non-targeted strand (NTS) and the targeted strand (TS) are marked with black triangles.

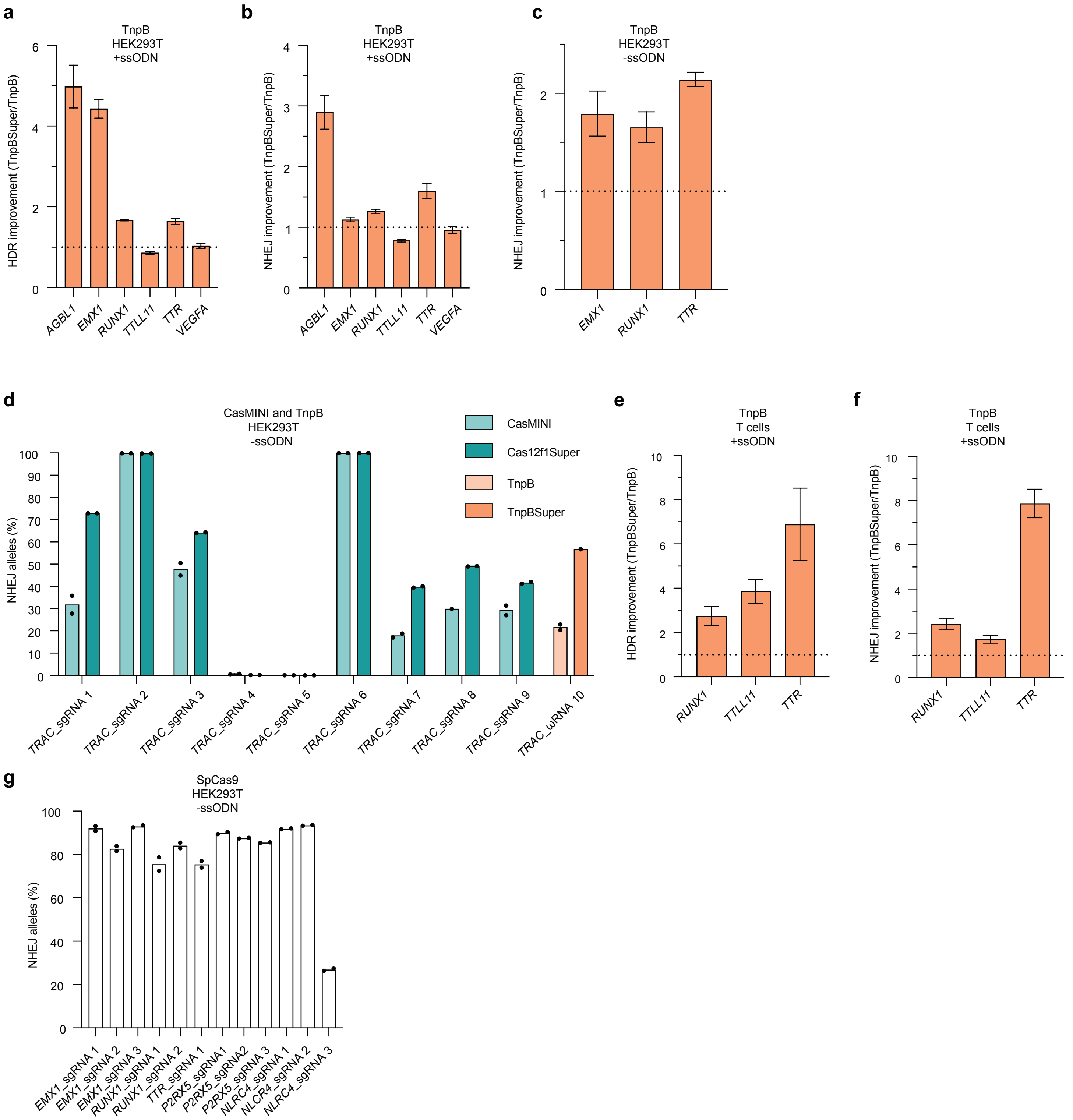

Fig. S8: *Improved variants Cas12f1Super and TnpBSuper edit endogenous genomic loci.* **a, b** Fold change of HDR efficiency (**a**) or NHEJ efficiency (**b**) of TnpBSuper relative to TnpB in presence of ssODN measured with ampliconNGS at indicated endogenous loci in HEK293T cells (*n* = 3 biological replicates). **c** Fold change of NHEJ efficiency of TnpBSuper relative to TnpB in absence of ssODN measured with ampliconNGS at indicated endogenous loci in HEK293T cells (*n* = 3 biological replicates). Bars represent mean ± standard deviation. Fold change was calculated from group means, with propagated standard deviation shown as error bars. **d** Absolute NHEJ efficiency of Cas12f1Super or TnpBSuper measured with ampliconNGS at indicated guides targeting *TRAC* locus in HEK293T cells compared to CasMINI or TnpB (*n* = 2 biological replicates). Each dot represents an individual biological replicate, and bars represent the mean. **e, f** Fold change of HDR (**e**) or NHEJ efficiency (**f**) of TnpBSuper relative to TnpB in the presence of ssODN

measured with ampliconNGS at indicated endogenous loci in primary T cells (*n* = 3 biological replicates). TnpBSuper or TnpB were electroporated as mRNA, along with synthetic ωRNA and ssODN. **g** Absolute NHEJ efficiency of SpCas9 in the absence of ssODN measured with ampliconNGS at indicated at indicated loci in HEK293T cells (*n* = 2 biological replicates). Each dot represents an individual biological replicate, and bars represent the mean.

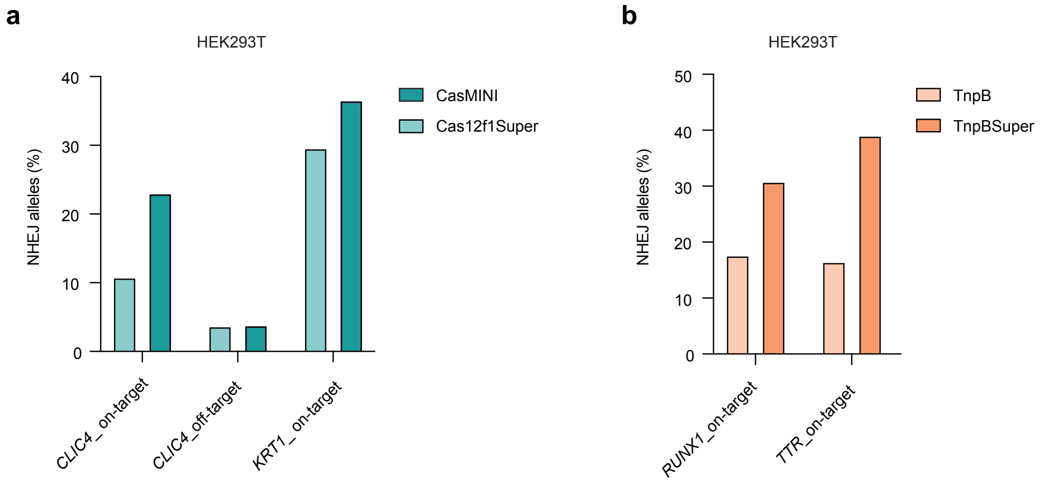

Fig. S9: *CasMINI/Cas12f1Super and TnpB/TnpBSuper NHEJ efficiency at on-target and off-target sites.* **a** NHEJ efficiency of CasMINI and Cas12f1Super measured with ampliconNGS at the indicated on-target and off-target sites in HEK293T cells. **b** NHEJ efficiency of TnpB and TnpBSuper measured with ampliconNGS at the indicated on-target sites in HEK293T cells. Bars represent the value of a single experimental replicate.

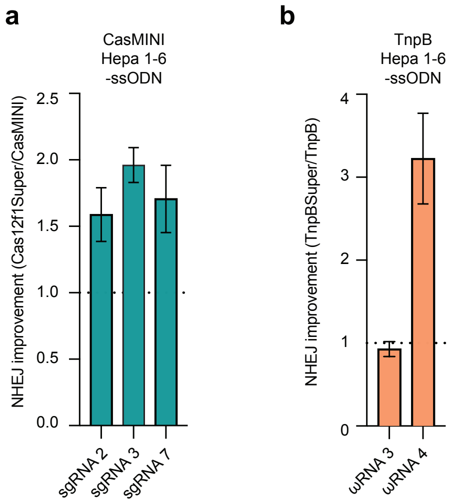

Fig. S10: *Improved variants Cas12f1Super and TnpBSuper edit endogenous genomic loci.* **a** Fold change of NHEJ efficiency of Cas12f1Super relative to CasMINI (**a**) or of TnpBSuper relative to TnpB (**b**) measured with ampliconNGS at indicated targets within the *Pcsk9* gene in Hepa 1-6 cells (n = 3 biological replicates). Each nuclease-guide RNA combination was delivered on a “all-in-one” plasmid. Bars represent mean ± standard deviation. Fold change was calculated from group means, with propagated standard deviation shown as error bars.

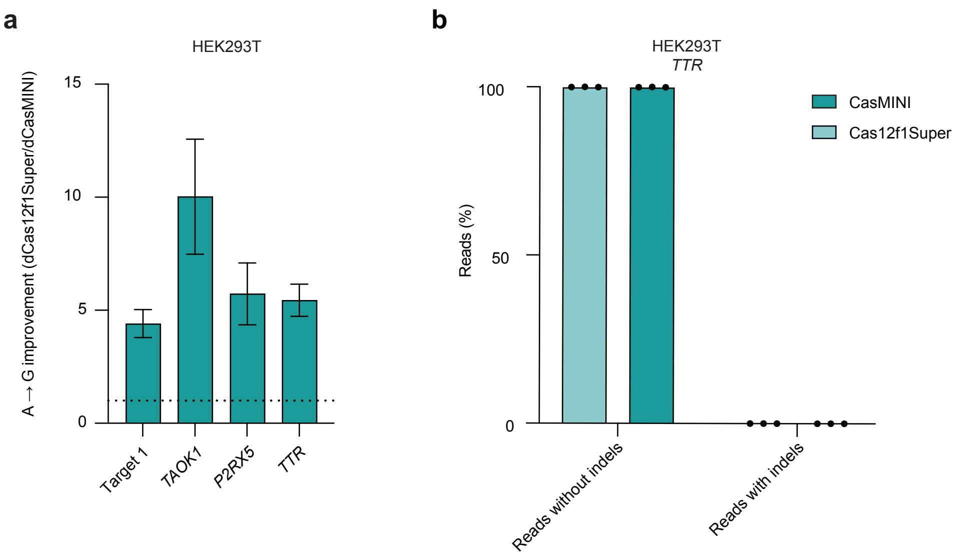

Fig. S11: *Improved variant dCas12f1Super outperforms dCasMINI as an adenine base editor.* **a** Fold change of overall adenine base editing activity of dCas12f1Super relative to dCasMINI measured with ampliconNGS at Target CM1 and indicated endogenous sites in HEK293T cells (*n* = 3 biological replicates). Bars represent mean ± standard deviation. Fold change was calculated from group means, with propagated standard deviation shown as error bars. **b** Frequency or reads without and with indels upon editing *TTR* site with adenine base editor based on dCasMINI or dCas12f1Super in HEK293T cells (*n* = 3 biological replicates). Each dot represents an individual biological replicate, and bars represent the mean.

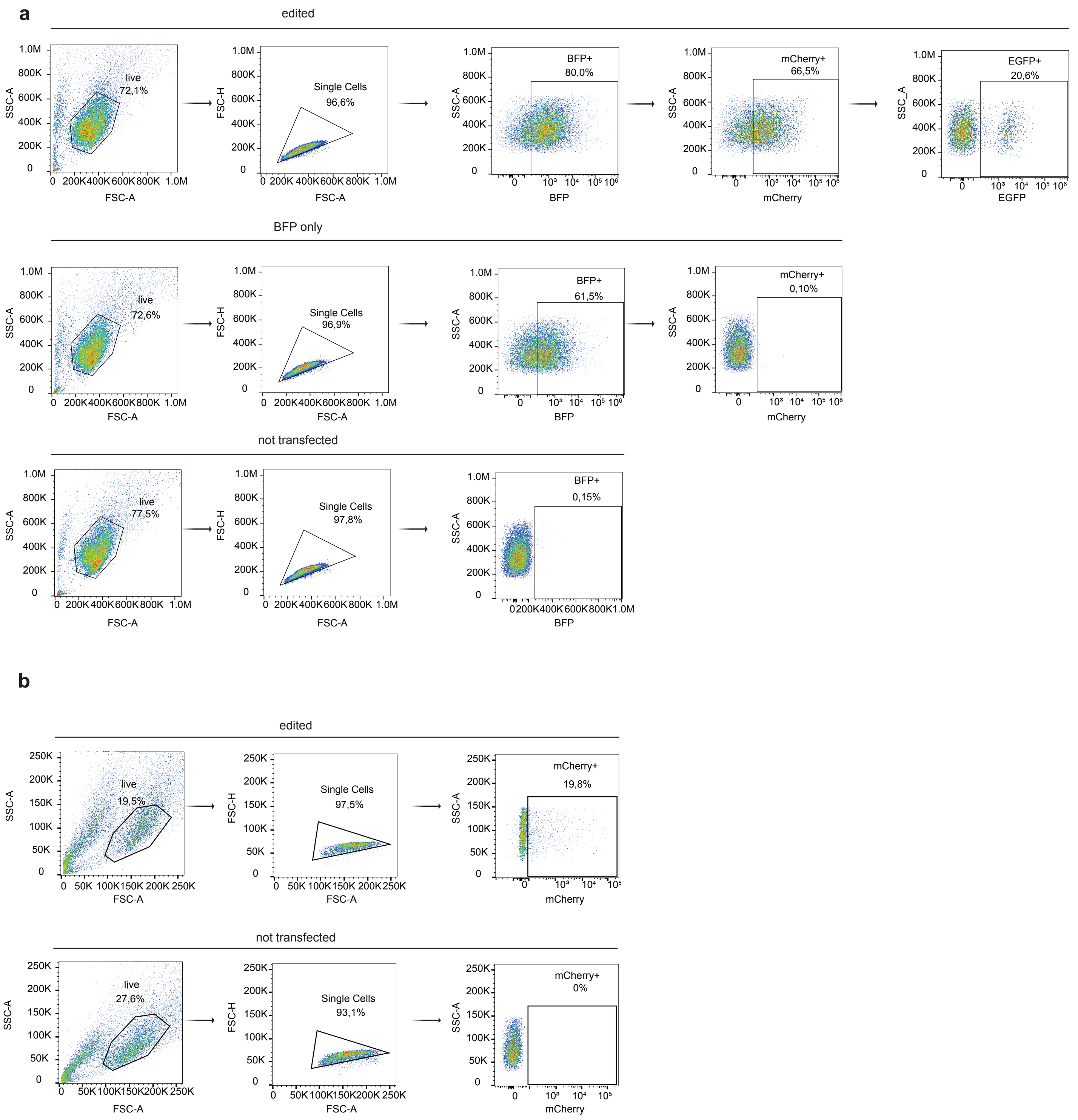

Fig. S12: *Gating strategies for flow cytometry and FACS experiments related to testing editing at HDR reporter targets and endogenous sites in HEK293T and Hepa 1-6 cell lines.* **a** Representative plots showing the gating strategy for measuring HDR at HDR reporter targets in HEK293T cell line. Cells were gated for live cells, singlets, BFP positivity, mCherry positivity (BFP marks the guide-expressing construct; mCherry marks the nuclease-expressing construct) and finally, for EGFP positivity. In case of “edited” sample one of the CasMINI variants was co-delivered with sgRNA for Target CM1 together with ssODN. “BFP” only and “not transfected” samples serve as controls for gating. In case of “BFP only” sample only sgRNA targeting Target CM1 was delivered. **b** Representative plots showing the gating strategy for FACS-sorting HEK293T or Hepa 1-6 cells for measuring editing. Cells were gated for live cells, singlets and mCherry positivity. In case of “edited” sample CasMINI and sgRNA were delivered on the same “all-in-one” plasmid. “Not transfected” sample serves as control for gating.

a

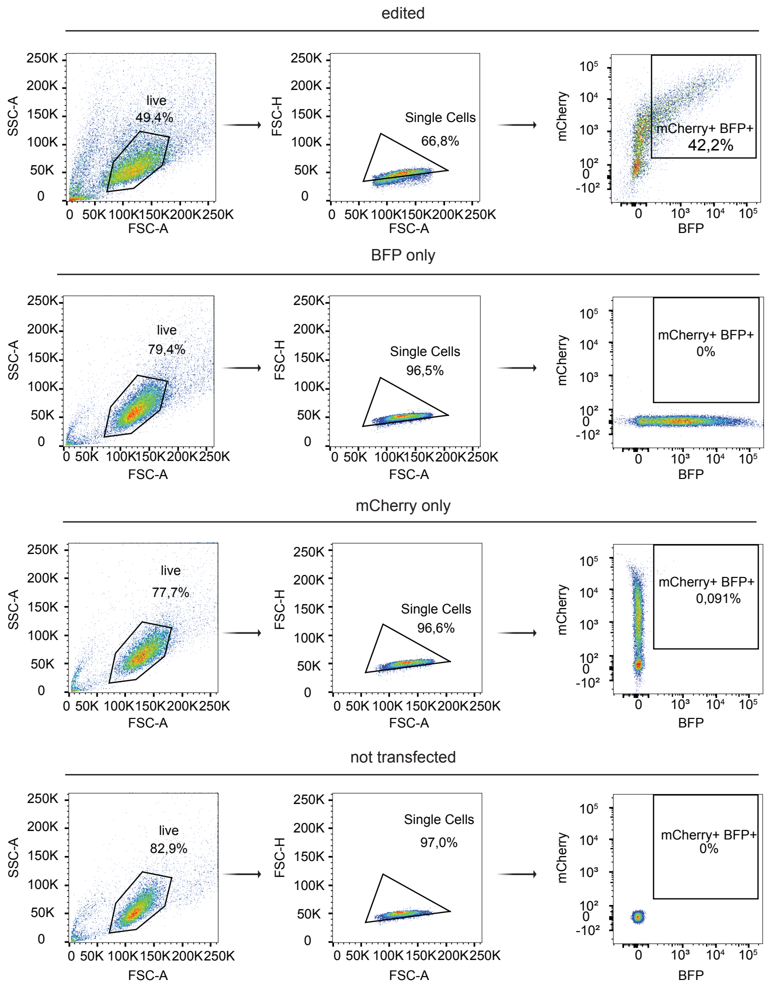
edited
live
49,4%
Single Cells
66,8%
mCherry+ BFP+
42,2%
BFP only
live
79,4%
Single Cells
96,5%
mCherry+ BFP+
0%
mCherry only
live
77,7%
Single Cells
96,6%
mCherry+ BFP+
0,091%
not transfected
live
82,9%
Single Cells
97,0%
mCherry+ BFP+
0%
SSC-A
FSC-A
FSC-H
mCherry
BFP

b

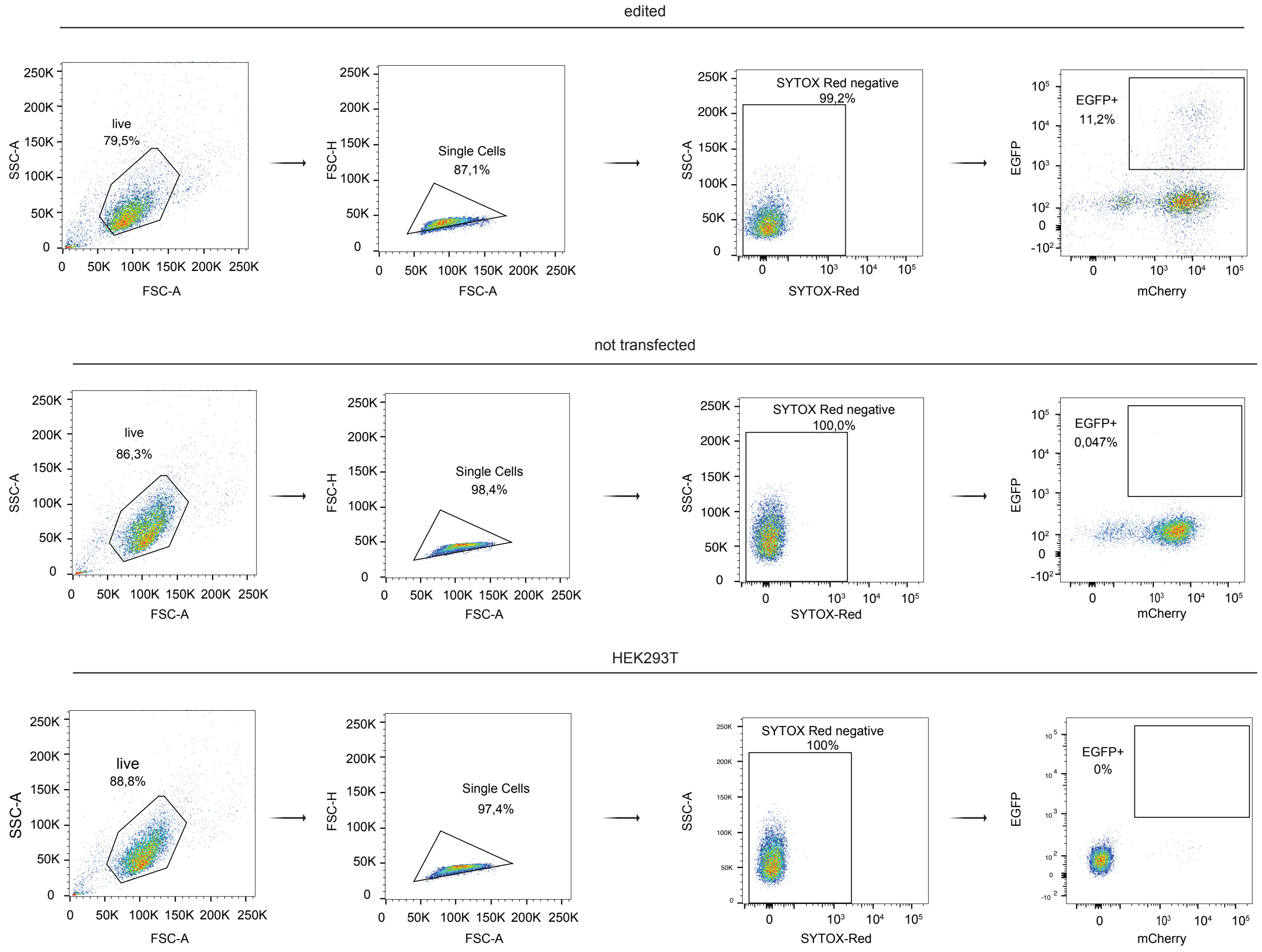
edited
live
79,5%
Single Cells
87,1%
SYTOX Red negative
99,2%
EGFP+
11,2%
not transfected
live
86,3%
Single Cells
98,4%
SYTOX Red negative
100,0%
EGFP+
0,047%
HEK293T
live
88,8%
Single Cells
97,4%
SYTOX Red negative
100%
EGFP+
0%
SSC-A
FSC-A
FSC-H
SYTOX-Red
EGFP
mCherry

Fig. S13: *Gating strategy for FACS experiments related to testing editing at endogenous sites in HEK293T and NIH-3T3 cell line and to big scale selection of CasMINI and TnpB for HDR in HEK293T cell line.* **a** Representative plots showing the gating strategy for FACS-sorting HEK293T and NIH-3T3 cell lines for measuring NHEJ at endogenous sites. Cells were gated for live cells, singlets and mCherry/BFP positivity (BFP marks the guide-expressing construct; mCherry marks the nuclease-expressing construct). In case of "edited" sample CasMINI was co-delivered with sgRNA and ssODN in HEK293T cells. "BFP only", "mCherry only" and "not transfected" samples serve as controls for gating. **b** Representative plots showing the gating strategy for FACS-sorting during round 1 of HDR selection of CasMINI in HEK293T-reporter cell line. Cells were gated for live cells, singlets, SYTOX-Red-negativity, and finally for mCherry/EGFP positivity. In case of "edited" sample sgRNA for Target CM1 was delivered together with ssODN. "Not transfected" and "HEK293T" samples serve as controls for gating. In case of "not transfected" control sgRNA and ssODN were not delivered.
