# Supplementary material for: Directed evolution of compact RNA-guided nucleases for enhanced activity in mammalian cells
Source: Genome Biol. 2026 Jun 16;27:213. doi: 10.1186/s13059-026-04144-5 (PMC13330202; doi:10.1186/s13059-026-04144-5)
Supplement: Supplementary file 3 — Additional file 3. Contains unprocessed western blots. [file 13059_2026_4144_MOESM3_ESM.pdf]

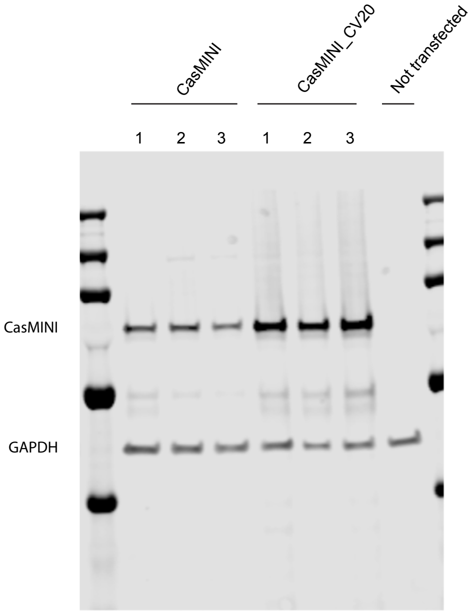

Unprocessed western blot for Additional file 1: Fig. S3d

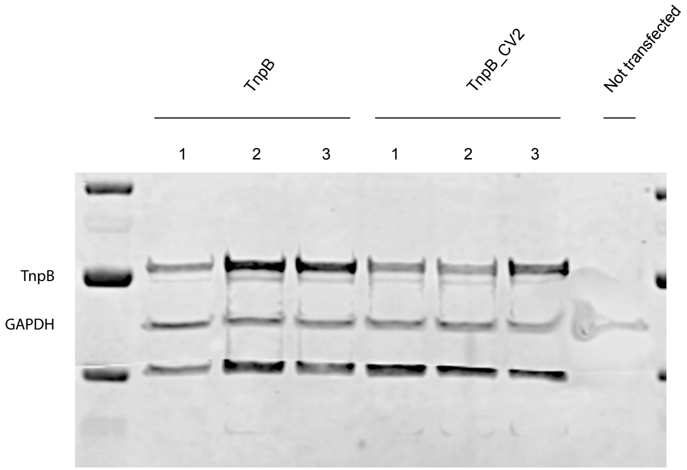

Unprocessed western blot for Additional file 1: Fig. S5c
